# Supplementary figures and images for: eDNA surveys substantially expand known geographic and ecological niche boundaries of marine fishes
Source: PLoS Biol. 2025 Oct 30;23(10):e3003432. doi: 10.1371/journal.pbio.3003432 (PMC12574855; doi:10.1371/journal.pbio.3003432)

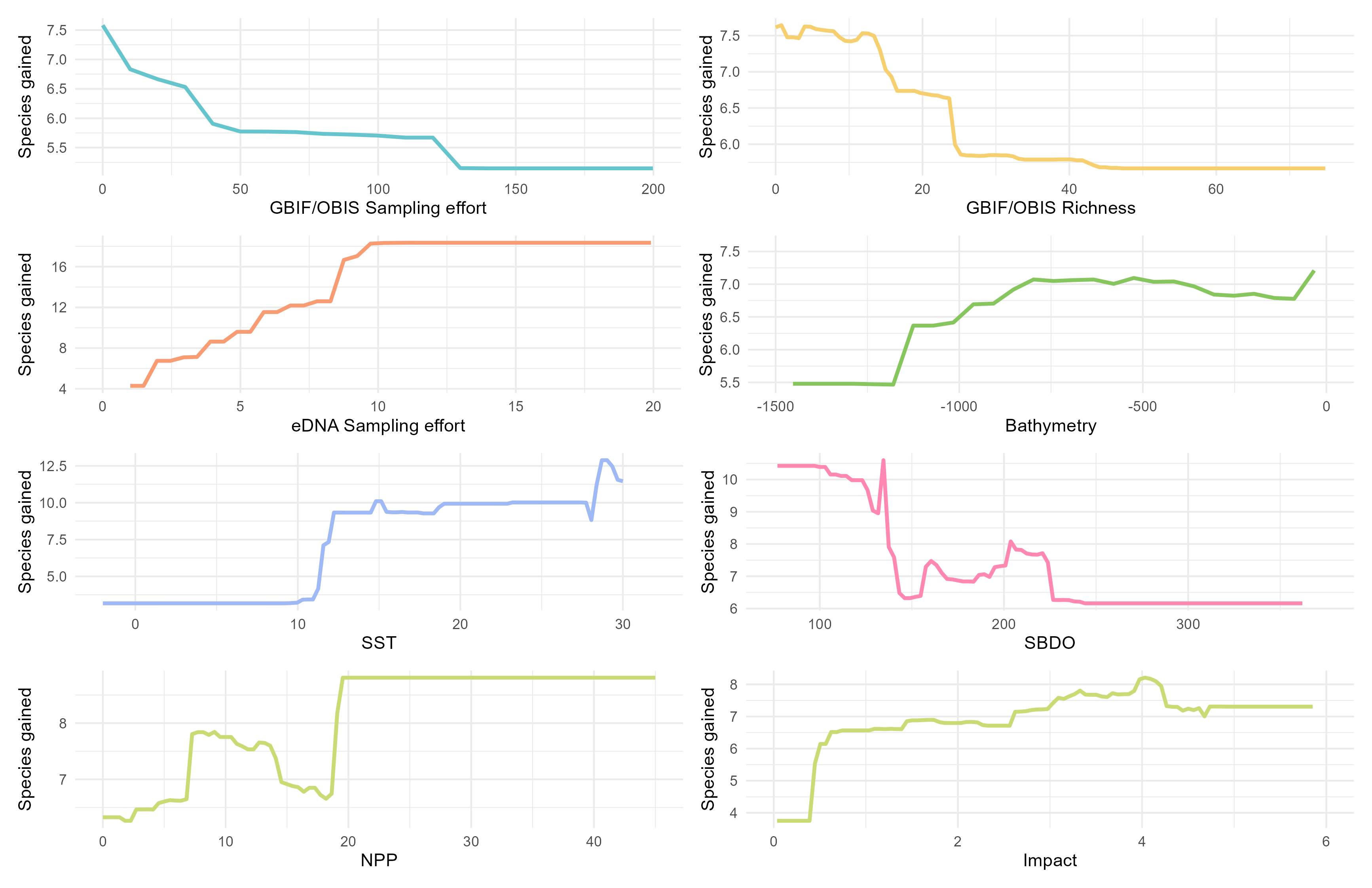

Supplement: S1 Fig — The data underlying this figure can be found in https://doi.org/10.6084/m9.figshare.30138472. (PNG) [file pbio.3003432.s001.png]

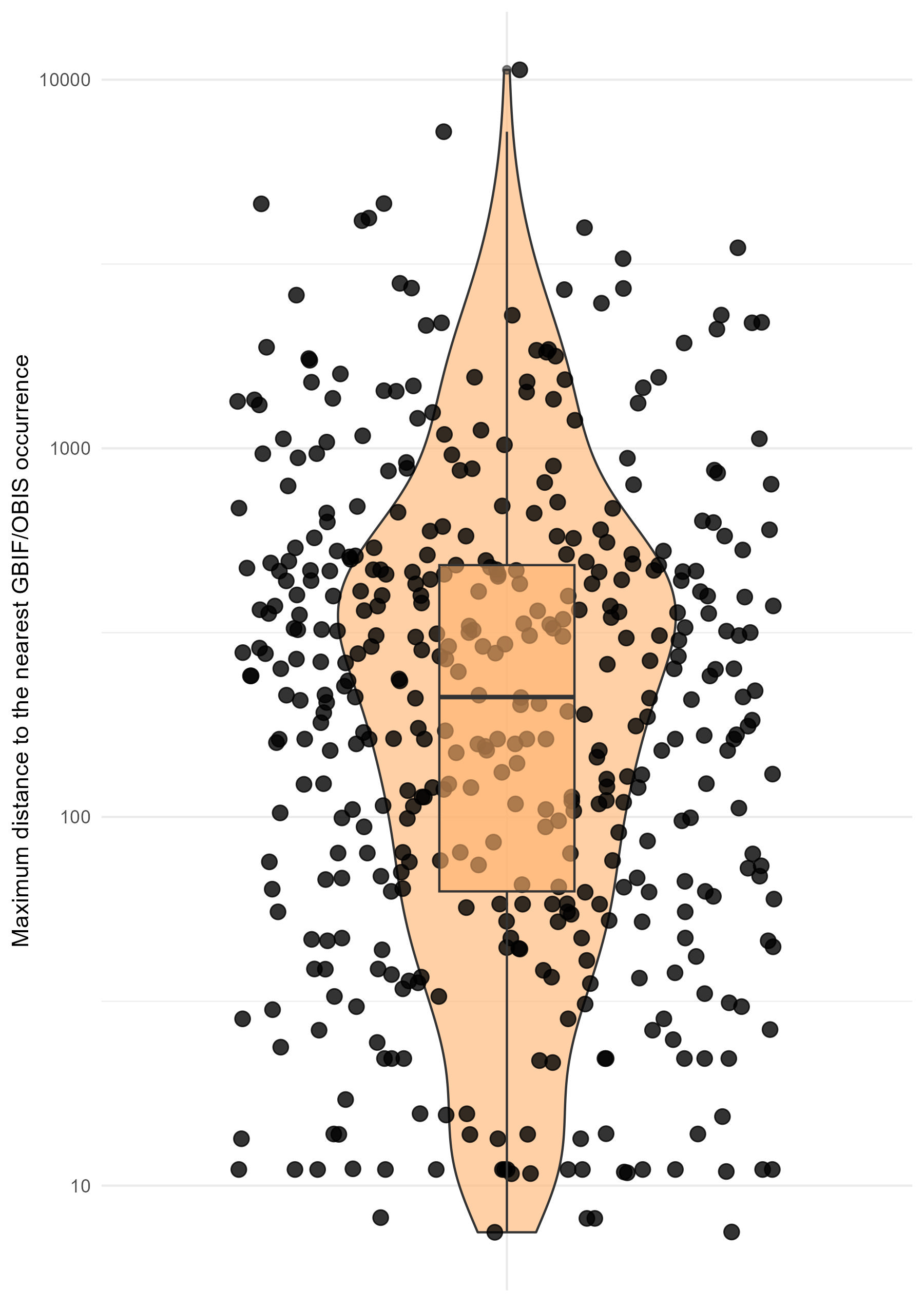

Supplement: S2 Fig — The data underlying this figure can be found in https://doi.org/10.6084/m9.figshare.30138277. (JPG) [file pbio.3003432.s002.jpg]

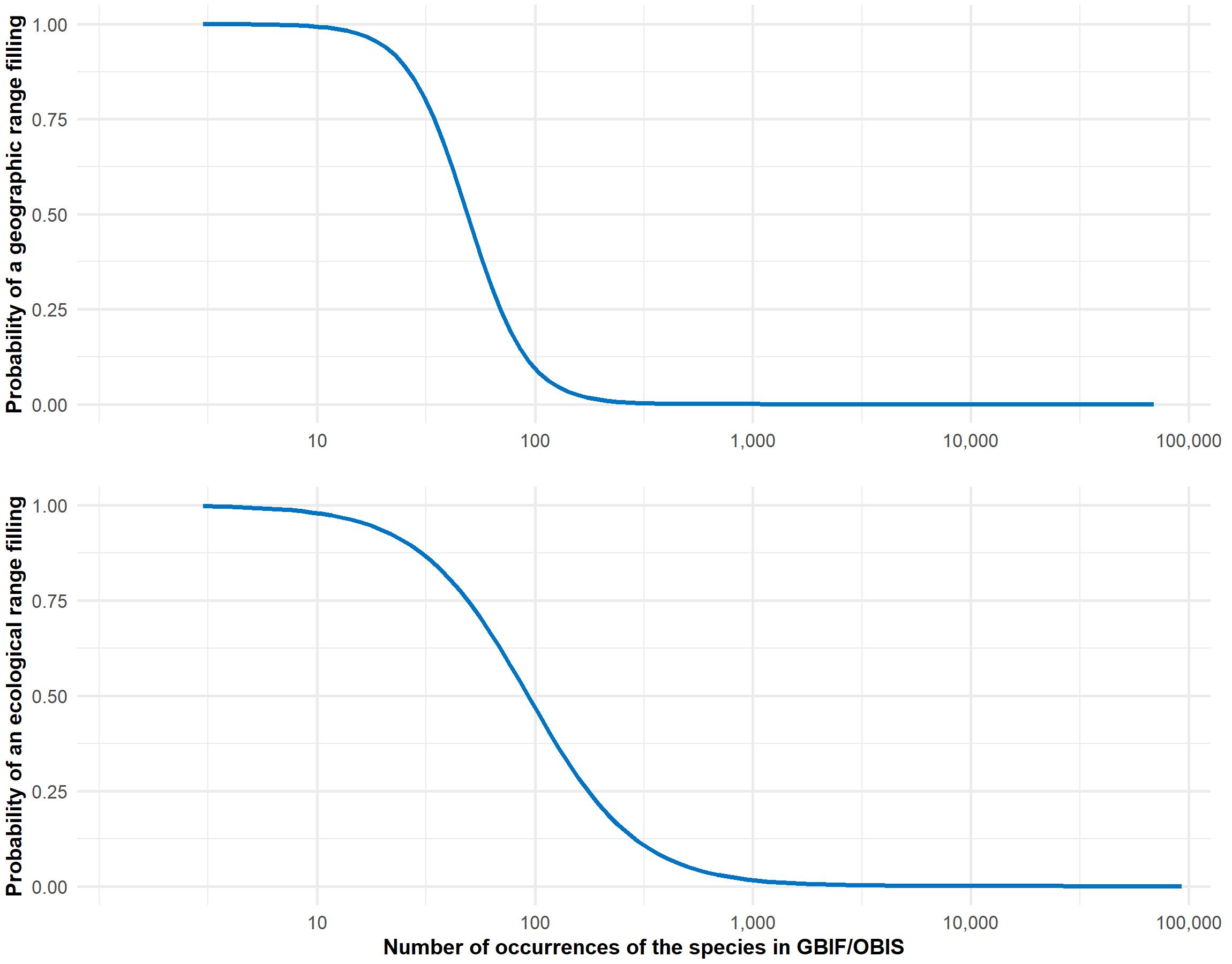

Supplement: S3 Fig — For ecological niche range fillings (b), as we have an over-representation of zeros in our dataset (10,952 out of 11,018), we randomly downsampled the dataset 1,000 times, to obtain 75% of zeros in the downsampled datasets. This relationship is estimated with the median coefficient of the 1,000 models realized with randomly sampled zeros. Therefore, the predicted probabilities do not represent absolute, realistic values but rather an estimated trend. The data underlying this figure can be found in https://doi.org/10.6084/m9.figshare.30146266. (JPEG) [file pbio.3003432.s003.jpeg]

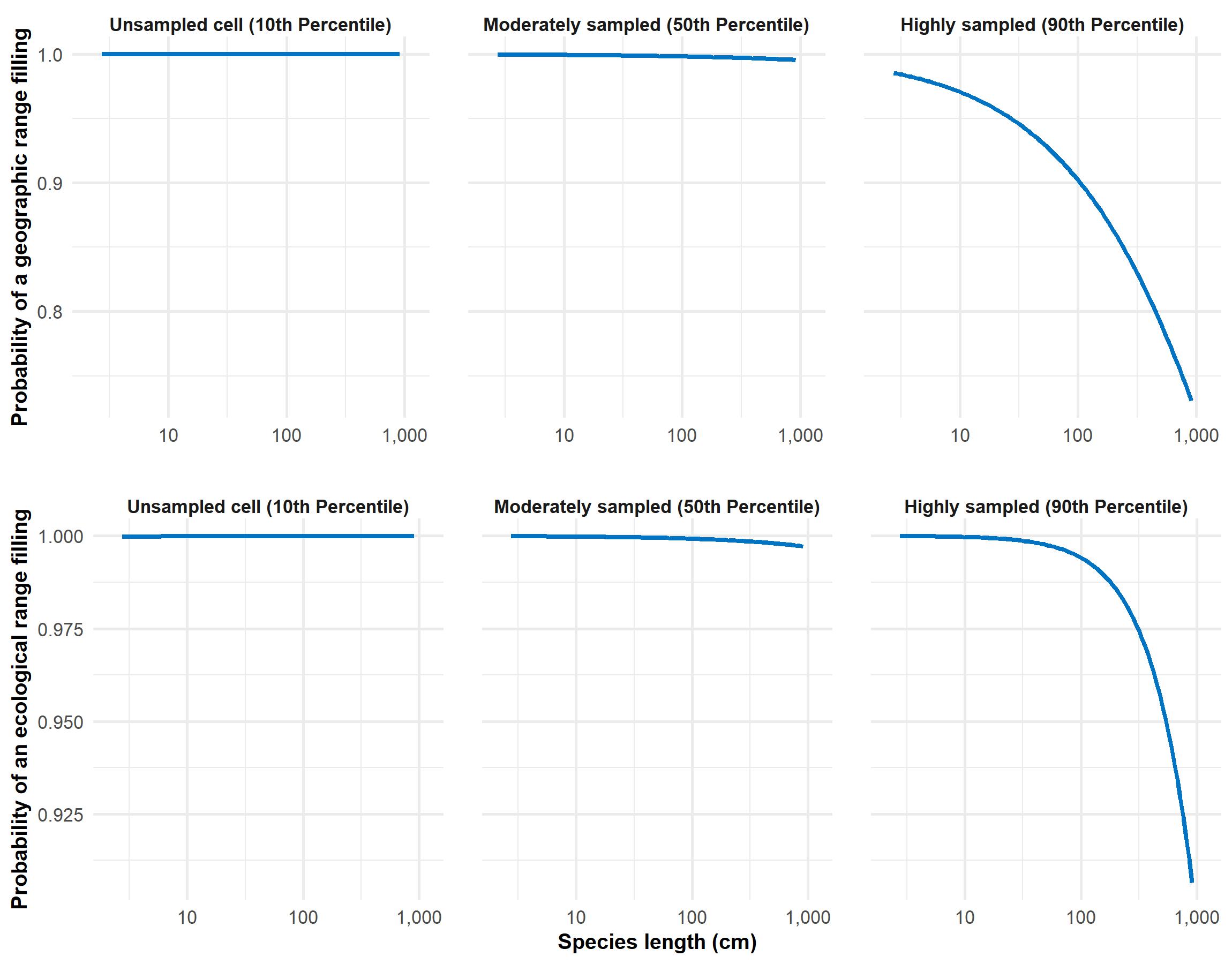

Supplement: S4 Fig — This relationship is represented for three different cell sampling intensities from unsampled (0 sampling events; 10th percentile), to moderately sampled (38 sampling events, 50th percentile), and highly sampled (354 sampling events, 90th percentile). As we have an over-representation of zeros in our dataset (10,952 out of 11,018), we randomly downsampled the dataset 1,000 times, to obtain 75% of zeros in the downsampled datasets. This relationship is estimated with the median coefficient of the 1,000 models realized with randomly sampled zeros. Therefore, the predicted probabilities do not represent absolute, realistic values but rather an estimated trend. The data underlying this figure can be found in https://doi.org/10.6084/m9.figshare.30146266. (JPEG) [file pbio.3003432.s004.jpeg]
