# Supplementary material for: eDNA surveys substantially expand known geographic and ecological niche boundaries of marine fishes
Source: PLoS Biol. 2025 Oct 30;23(10):e3003432. doi: 10.1371/journal.pbio.3003432 (PMC12574855; doi:10.1371/journal.pbio.3003432)
Supplement: S1 Table — Checklists of sampling areas and neighboring regions were used to compute the coverage. (DOCX) [file pbio.3003432.s005.docx]

| **Project name** | **Sampling Area** | **Neighboring provinces** |
| --- | --- | --- |
| Svalbard | Svalbard | Arctic |
| Arctic | Greenland | Arctic |
| Shift-eDNA | Baffin Sea | Arctic  Cold Temperate Northwest Atlantic |
| UP2023 | Channel and Fjords of Southern Chile | Magellanic  Scotia Sea  Warm Temperate Southeastern Pacific |
| Antarctic | Continental High Antarctic | Magellanic  Scotia Sea |
| med | Mediterranean Sea | - |
| atlantic | Northern European Seas | Lusitanian |
| Macobios | Tropical Northwestern Atlantic | - |
| TacDNA | Western Indian Ocean | - |
| Zamacos | Western Indian Ocean | - |
| LaPerouse | Western Indian Ocean | - |
| fakarava | Central Polynesia | Southeast Polynesia  Marquesas |
| malpelo | Tropical East Pacific | Galapagos |
| lengguru | Western Coral Triangle | Sahul Shelf |
| Curaçao | Tropical Northwestern Atlantic | - |
| Caraïbes | Tropical Northwestern Atlantic | - |
