# Supplementary material for: eDNA surveys substantially expand known geographic and ecological niche boundaries of marine fishes
Source: PLoS Biol. 2025 Oct 30;23(10):e3003432. doi: 10.1371/journal.pbio.3003432 (PMC12574855; doi:10.1371/journal.pbio.3003432)
Supplement: S2 Table — (DOCX) [file pbio.3003432.s006.docx]

| **Species** | **Family** |
| --- | --- |
| *Isurus oxyrinchus* | Lamnidae |
| *Lamna nasus* | Lamnidae |
| *Carcharhinus obscurus* | Carcharhinidae |
| *Alopias pelagicus* | Alopiidae |
| *Alopias vulpinus* | Alopiidae |
| *Alopias superciliosus* | Alopiidae |
| *Odontaspis ferox* | Odontaspididae |
| *Mobula mobular* | Myliobatidae |
| *Mobula thurstoni* | Myliobatidae |
| *Mobula birostris* | Myliobatidae |
| *Mobula tarapacana* | Myliobatidae |
