# Supplementary material for: eDNA surveys substantially expand known geographic and ecological niche boundaries of marine fishes
Source: PLoS Biol. 2025 Oct 30;23(10):e3003432. doi: 10.1371/journal.pbio.3003432 (PMC12574855; doi:10.1371/journal.pbio.3003432)
Supplement: S3 Table — (DOCX) [file pbio.3003432.s007.docx]

| **Species** | **Location** | **Reason** |
| --- | --- | --- |
| *Anampses chrysocephalus* | Lengguru, West Papua | Endemic to Hawaii |
| *Merluccius productus* | La Pérouse Mount, La Réunion | Consumption |
| *Merluccius merluccius* | Pond Inlet, Canada | Consumption |
| *Melanogrammus aeglefinus* | Pangnirtung, Canada | Consumption |
| *Sardinella longiceps* | Corsica, France | Consumption |
| *Chelon ramada* | Curacao, Caribbean | 12 reads |
| *Encrasicholina punctifer* | Marseille, France | Consumption (58 reads) |
| *Sparisoma viride* | Lengguru, Indonesia | Contamination |
| *Sparisoma aurofrenatum* | Lengguru, Indonesia | Contamination |
| *Sparisoma chrysopterum* | Lengguru, Indonesia | Contamination |
