# Supplementary material for: eDNA surveys substantially expand known geographic and ecological niche boundaries of marine fishes
Source: PLoS Biol. 2025 Oct 30;23(10):e3003432. doi: 10.1371/journal.pbio.3003432 (PMC12574855; doi:10.1371/journal.pbio.3003432)
Supplement: S1 Methods — (DOCX) [file pbio.3003432.s008.docx]

**Supporting methods**

*DNA extraction*

DNA extraction was carried out in a dedicated laboratory designed for water DNA sample processing, featuring positive air pressure, UV treatment, and frequent air renewal to minimize contamination. Before entering, personnel changed into full protective clothing, including a disposable bodysuit with a hood, a mask, laboratory shoes, overshoes, and gloves, in a designated transition area. Work surfaces were decontaminated with 10% commercial bleach before and after each procedure. For extraction, filtration capsules containing CL1 buffer were agitated for 15 minutes at 800 rpm on an S50 shaker (cat Ingenieurbüro™). The buffer was then transferred to a 50-mL tube and centrifuged at 15,000 × g for 15 minutes. After centrifugation, the supernatant was carefully removed using a sterile pipette, leaving 15 mL of liquid in the tube. To this, 33 mL of ethanol and 1.5 mL of 3M sodium acetate were added, and the tubes were stored at -20°C overnight. Samples were then centrifuged again at 15,000 × g for 15 minutes at 6°C, and the supernatants were discarded. Following centrifugation, 720 μL of ATL buffer (DNeasy Blood & Tissue Extraction Kit, Qiagen) was added to the pellet, and the mixture was vortexed. The supernatant was transferred to 2-mL tubes containing 20 μL of Proteinase K and incubated at 56°C for two hours. DNA extraction was then performed using the NucleoSpin Soil kit (MACHEREY-NAGEL GmbH & Co., Düren, Germany), beginning from step 6 of the manufacturer’s protocol. Elution was carried out by adding 100 μL of SE buffer twice. Following extraction, DNA samples were tested for PCR inhibition using qPCR. If inhibition was detected, samples were diluted fivefold before amplification.

*DNA amplification*

DNA amplification was conducted in a final reaction volume of 25 μL, using 3 μL of DNA extract as the template. The reaction mixture included 1 U of AmpliTaq Gold DNA Polymerase (Applied Biosystems, Foster City, CA), 10 mM Tris-HCl, 50 mM KCl, 2.5 mM MgCl₂, 0.2 mM of each dNTP, 0.2 μM “teleo” primers (Valentini *et al.* 2016), 4 μM human blocking primer specific to the “*teleo*” primers, and 0.2 μg/μL bovine serum albumin (BSA, Roche Diagnostic, Basel, Switzerland). The “*teleo*” primers were 5’-labeled with an eight-nucleotide tag unique to each PCR replicate, ensuring at least three nucleotide differences between any two tags to facilitate sequence assignment during analysis. Identical tags were used for both forward and reverse primers within each replicate.

PCR amplification was carried out with an initial denaturation at 95°C for 10 minutes, followed by 50 cycles of 30 seconds at 95°C, 30 seconds at 55°C, and 1 minute at 72°C, with a final elongation step at 72°C for 7 minutes. Amplifications were performed in a dedicated facility for amplified DNA, maintained under negative air pressure and physically separated from the DNA extraction area, which was maintained under positive air pressure. Each filtration was subjected to 12 replicate PCRs, totaling 24 replicates per sampling site.

Following amplification, capillary electrophoresis (QIAxcel; Qiagen GmbH) was used to assess DNA quantity, after which the samples were purified with the MinElute PCR purification kit (Qiagen GmbH). Purified DNA was subsequently titrated again via capillary electrophoresis before sequencing. Equal volumes of purified PCR products were pooled to achieve a theoretical sequencing depth of 500,000 reads per sample.

PCR purification was conducted in a separate, dedicated room for amplified DNA analysis, maintained under negative air pressure and physically isolated from the eDNA extraction area. Work surfaces were decontaminated with 10% commercial bleach before and after each procedure to minimize contamination.

*DNA sequencing*

Library preparation and sequencing were performed by Fasteris (Geneva, Switzerland) using the MetaFast protocol (Fasteris, <https://www.fasteris.com/dna/?q=content/metafast-protocol-amplicon-metagenomic-analysis>). Paired-end sequencing (2 × 125 bp) was conducted on an Illumina HiSeq 2500 sequencer (Illumina, San Diego, CA, USA) using the HiSeq SBS Kit v4 (Illumina, San Diego, CA, USA), following the manufacturer’s guidelines.

A total of eleven libraries were sequenced in a single HiSeq run. To monitor potential contamination, nine negative extraction controls and ten negative PCR controls (ultrapure water, 12 replicates) were amplified and sequenced alongside the samples.
