# Supplementary material for: eDNA surveys substantially expand known geographic and ecological niche boundaries of marine fishes
Source: PLoS Biol. 2025 Oct 30;23(10):e3003432. doi: 10.1371/journal.pbio.3003432 (PMC12574855; doi:10.1371/journal.pbio.3003432)
Supplement: Attachment — Submitted filename: Response to reviewers - PLoS Biology.docx [file pbio.3003432.s009.docx]

**Response to reviewers - PLoS Biology**

Dear Editor,

We are very grateful to the Editor and Reviewers for their constructive comments, and we are pleased to submit the revised version of our manuscript entitled “The uncharted geographic and ecological niche boundaries of marine fishes”.

Overall, our review focused on two key aspects:

- Clarifying and better defining our framework, particularly to prevent misunderstandings about temporal range expansions. For this, we added and discussed a new Figure 2 describing our approach. We also replaced the term “range expansions” by “range completions.”
- Conducting more in-depth analyses to:
  - quantify the probability to detect completions due to new sampling locations and due to eDNA metabarcoding
  - quantify which range completions were due to a sampling effect

You can find the answers to all comments made by reviewers below (line numbers refer to the revised manuscript), with code RX#Y for reviewer X comment Y). Thank you for your consideration and we look forward to working with you during the editorial process.

All authors have seen and approved the revised version.

Yours sincerely,

Loïc Sanchez on behalf of all authors

**Editor :**

You'll see that reviewer #1 is broadly positive about the study, but thinks that you need to run deeper analyses to disentangle the two main issues (sampling location, and eDNA vs other occurrence data), to explore the effect of the number of eDNA occurrences, and to make some sort of geographically based cost-benefit analysis to help prioritise future sampling (my interpretation). Reviewer #2 is very positive, but also raises some key issues about DNA transport, robustness to spatial resolution, and taxonomic accuracy. Reviewer #3 is very critical, and we note that his/her tone is rather strong (for example, we do not think that you claim to show real temporal range expansions); however, s/he raises potentially addressable issues with the clarity of the manuscript and Figures, the coverage of the literature, and the failure to use Species Distribution Models.

Based on these reviews, I regret that we will not be able to invite revision of the study, as successfully addressing the concerns raised would entail a significant amount of work (beyond what could be reasonably expected of a revision) and whether the current conclusions would be supported is unclear. However, between them, the reviewers make some constructive suggestions, and we might be open to a future submission along the lines requested by reviewer #1, taking a deeper and more comprehensive analysis of the data, and addressing all concerns raised by the three reviewers.

Because performing the required analyses would require substantial cost and effort with an uncertain outcome, we cannot in good faith invite revision of your study. However, given our and the reviewers' interest in the topic, if further work were to allow you to comprehensively address the reviewer concerns outlined above, we would be willing to consider an extensively revised manuscript as a re-submission. The article would receive a new number and submission date and we would evaluate it in view of any related work published in the interim (applying our 6-month scooping protection policy, of course), but we would make every attempt to engage the same Academic Editor and reviewers in its assessment, and the process would not start from scratch but continue from here.

We appreciate that the scale of the requested additional work is significant and understand that you may prefer to pursue faster publication of this work elsewhere. At this point, your manuscript is no longer under active consideration at PLOS Biology. We might be able to facilitate a faster consideration at another PLOS journal; if this is of interest to you, please let me know. Please feel free to contact me if you would like to discuss next steps for your manuscript. If you do make the choice to revise and re-submit to PLOS Biology, please provide a point-by-point response to the reviewers' comments when re-submitting. You should include this as a separate file, labelled 'Response to Reviewers'. Please also provide the tracking number of this manuscript [PBIOLOGY-D-24-02395R1] in the 'previous interaction' section of the online submission form and in your cover letter.

I am sorry that we cannot be more positive on this occasion. I thank you for having considered PLOS Biology for publication and hope that you will find the reviewers' feedback helpful as you consider how to proceed with this work.

**Reviewer #1 (Jonathan Belmaker):**

This study uses a comprehensive and impressive global eDNA sampling campaign to show that the current environmental and geographic niche, as estimated by GBIF and OBIS, is underestimated for a vast majority of species. The actual results are not surprising, as biases in such GBIF and OBIS occurrences records are well documented. However, the sheer scale of the analyses presented here make this a strong case for the need for more, and better, sampling. Thus, I feel this study has potential and should be published. However, I do have a few major comments that I feel could be used to improve this study:

Thank you for this overall positive assessment, we did our best to address your comments. As an introductory response, we would like to mention that we replaced “range expansions” by “range completions” throughout the manuscript, in order to avoid any confusion with temporal species range shifts.

R1#1 I feel there are two major points in this manuscript, and that currently they are entangled. The first is the importance of sampling locations, which were chosen to be dispersed and very different from the occurrence records in GBIF and OBIS. The take home message here is that deliberate dispersed sampling may have strong advantages in better estimating species niches. The second is the use of eDNA versus open source occurrence data. Here, eDNA recovered more species, even in locations where occurrence records were present. This is shown by the detection of smaller fishes with eDNA. However, the exact contribution of each of these (sampling location versus sampling method) is not quantified. I think that deeper analyses, e.g., use of some kind of null model to disentangle these separate contributions, and then discussion of this issue is warranted.

R1#1 We fully agree with the reviewer that there was room for improving our framework and take-home messages. The main message that we want to highlight in this paper is that species geographic ranges and ecological niches (encompassing an anthropogenic dimension) are biased and that both the sampling of new remote locations and the use of eDNA metabarcoding in previously sampled locations can mitigate such biases.

Yet, our explanation on the distinction between new detections attributed to eDNA methodology and those attributed to sampling in remote or unsampled locations was not clear while being a crucial point of our study. We now clearly distinguish new detections in cells previously sampled in GBIF/OBIS and new detections in cells that were never reported in GBIF/OBIS. We consider that in the former case, new detections address a methodological bias since fish species were missed in previous surveys, while in the latter case, new detections address a geographic bias since these cells had not previously been sampled, at least for fish. To illustrate this central point, we included a new figure in the introduction (Fig. 2; see hereunder) associated with a better definition of the framework distinguishing methodological and geographic sampling biases. It also describes how we quantified geographic and ecological range completions. We also added a sentence in the abstract and clarified the whole framework in the manuscript L133:

[Aggregating all this data with our eDNA samples allowed us to reveal geographic and ecological range completions as described in Fig. 2. Range completions detected by eDNA in cells that had previously been sampled in GBIF/OBIS address a methodological sampling bias, since the species had been missed in the projects that reported the data. However, we consider that range completions detected by eDNA in cells that had never been sampled address the geographic sampling bias, since we cannot quantify the extent to which eDNA facilitated the detection in this case.]

*Figure 2: Description of the framework used in the analyses. (a) Example of species occurrences in GBIF/OBIS. New eDNA species occurrences (orange) were added to existing GBIF/OBIS occurrences (black). (b) geographic range completions were measured as the maximum distance between new eDNA occurrences and the nearest GBIF/OBIS occurrence. (c) Ecological range completions were measured as the increase in the ecological niche breadth of each species, based on the minimum and maximum values encountered before and after updating the niche with eDNA occurrences.*

Regarding the quantification of both sampling biases (methodological and geographic), as we already quantified whether each detection was due to eDNA method or new sampling locations, we are also able to answer the reviewer’s concern: “eDNA recovered more species, even in locations where occurrence records were present”, since we mention that 65% of the ecological range completions were found by addressing the methodological sampling bias. It appears that Figure 5 was not clear enough, so we modified it.

To provide more quantitative information, we included two new analyses - one for each type of range completions (*i.e.,* geographic and ecological). First, we modeled the probability to detect a range completion for any eDNA detection in a given cell depending on species size, total number of species occurrences in GBIF/OBIS and GBIF/OBIS sampling intensity in the cell. We show a similar pattern for geographic and ecological range completions: the probability to detect a species range completion is higher in unsampled cells for all fish body sizes (addressing the geographic sampling bias). However, in highly sampled cells, the probability to detect a species range completion depends on body size with small-bodied fishes being detected in cells having many sampling events (addressing the methodological bias). We included these results in two supplementary figures Suppl. Fig 2 and 3, presented hereunder with their captions and discussed them in the main document (L221):

[We then used logistic regressions to quantify the probability of detecting geographic or ecological niche range completions for any eDNA detection, depending on species body size, total number of species occurrences on GBIF/OBIS, and GBIF/OBIS sampling intensity of the cell. The models show consistent trends for both types of completions (S2-3 Figures). The probability of detecting a range completion is higher in unsampled cells (addressing the geographic sampling bias), while in already sampled cells, the probability depends on fish body length, as we are still able detect range completions for small-bodied fish in highly sampled cells (addressing the methodological sampling bias). Detections of species with a lower number of occurrences have a significantly higher probability of displaying a range completion, indicating that some of the completions we found may only be the result of a sampling effect. We used a null model to test if our eDNA samplings contributed to ecological range completions more than what would be expected by adding new random GBIF/OBIS occurrences (see Methods). The null model indicated that 32 out of 66 ecological range completions were due to a sampling effect, *i.e.* just randomly adding a certain number of samples, while the 34 remaining completions were due to addressing either the geographic or the methodological sampling bias. A specific gobiid, the coralline goby (*Odondebuenia balearica*), notably had more occurrences in our new eDNA samples than reported in GBIF/OBIS.]

Supplementary Figure 2: Predicted probability of a species detection being a geographical (a) or ecological (b) range completion as a function of the number of occurrences in GBIF/OBIS, while setting the sampling intensity and body length at their median value. For ecological range completions (b), as we have an over-representation of zeros in our dataset (10,952 out of 11,018), we randomly downsampled the dataset 1,000 times, to obtain 75% of zeros in the downsampled datasets. This relationship is estimated with the median coefficient of the 1,000 models realized with randomly sampled zeros. Therefore, the predicted probabilities do not represent absolute, realistic values but rather an estimated trend.

Supplementary Figure 3: Predicted probability of a species detection being a geographical (a) or ecological (b) range completion as a function of the body length of the fish species in cm, while setting the number of species occurrences in GBIF/OBIS at its median value. This relationship is represented for three different cell sampling intensities from unsampled (0 sampling events; 10th percentile), to moderately sampled (38 sampling events, 50th percentile), and highly sampled (354 sampling events, 90th percentile). As we have an over-representation of zeros in our dataset (10,952 out of 11,018), we randomly downsampled the dataset 1,000 times, to obtain 75% of zeros in the downsampled datasets. This relationship is estimated with the median coefficient of the 1,000 models realized with randomly sampled zeros. Therefore, the predicted probabilities do not represent absolute, realistic values but rather an estimated trend.

R1#2 The authors acknowledge that niche expansion estimates will depend on the number of occurrences (for under sampled species each new occurrence will likely expand the niche). However, this is never actually accounted for or quantified. Here too, I feel the use of some kind of null model to show how much each new eDNA occurrence contributes to niche expansion beyond what would be expected from another occurrence for that particular species (potentially separately estimated within or outside the estimates species range, e.g., using a convex hull) will make this manuscript a lot stronger.

R1#2 Thank you for the suggestion. We set up a null model to test if our eDNA samples, whether they address a methodological or a geographic bias, contribute to ecological range completions beyond what would be expected from adding new GBIF/OBIS occurrences randomly. In other words, we tested whether species range completions were due to a sampling effect, *i.e.* adding a certain number of samples, or to new sampling effort towards certain overlooked locations combined to eDNA methodology.

We generated 1,000 samples for each species, that were randomly selected among their total number of GBIF/OBIS occurrences. More precisely, we randomly selected a subset by removing a number of GBIF/OBIS occurrences (*N*), where *N* corresponds to the number of new eDNA occurrences for a given species. For each of these random samples, we then computed the ecological range completion after adding the *N* randomly removed GBIF/OBIS occurrences. This produced a null distribution of expected range completions under the assumption that adding *N* new GBIF/OBIS random occurrences (without our eDNA samplings) changes the perceived species ranges. We then compared our observed range completion (calculated when adding our eDNA occurrences) to this null distribution. We found that for 31 out of 62 range completions, the observed completion was in the top 5% of the expected values. This suggests that observed range completions with eDNA samples were significantly larger than expected from random sampling GBIF/OBIS occurrences alone. The remaining 31 completions fell within the expected range, meaning they could be the result of simply adding new samples.

However, a key limitation of our null model is that it only generates random occurrences within previously sampled locations. Since our eDNA samples occur also in locations where no data was available, the model cannot generate random occurrences in these unsampled GBIF/OBIS locations. As a result, when eDNA contributes to range completions in previously unsampled regions, we cannot determine whether this effect is due to eDNA’s unique ability to detect species or simply because we sampled a new overlooked area. We added the null model in the methods, and the results L229:

[We used a null model to test if our eDNA samplings contributed to ecological range completions more than what would be expected by adding new random GBIF/OBIS occurrences (see Methods). The null model indicated that 32 out of 66 ecological range completions were due to a sampling effect, *i.e.* just randomly adding a certain number of samples, while the 34 remaining completions were due to addressing either the geographic or the methodological sampling bias. A specific gobiid, the coralline goby (*Odondebuenia balearica*), notably had more occurrences in our new eDNA samples than reported in GBIF/OBIS. ]

R1#3 I feel a quantification of the advantages, in terms of niche expansion estimates and actual estimates expenses (i.e., in $), of additional (a) non-eDNA surveys in remote location (b) eDNA surveys in remote locations (c) eDNA surveys in already sampled location will substantially enhance this study. I envision some kind of geographic layer of sampling priorities. This will move this study from one that points at kind-of-obvious conclusions into something that is both novel and useful.

Since the extent of eDNA methodology's contribution to species detections in remote locations remains unknown compared to classical surveys, we cannot really assess the benefits of eDNA surveys in remote locations. Yet, since eDNA detects a wide spectrum of species, we would recommend its use in any case, to maximize the chances to detect new species occurrences, in complementarity with more classical sampling techniques like underwater visual counts or baited video surveys (Polanco *et al.*, 2021; Marques *et al.*, 2021; Muenzel *et al.*, 2024, West *et al.*, 2024). In addition, estimating the cost of eDNA metabarcoding in specific locations would depend on several aspects: sampling method (niskin, peristaltic pump, underwater drone *etc.* Alexander *et al.*, 2023), sequencing depth, number of replicates (Stauffer *et al.*, 2021), number of markers used in the analysis, number of taxa added to the genetic reference database *etc.* As a consequence, estimating the time and funding efforts between methods seems out of the scope and could be the goal of a separate paper.

R1#4 I acknowledge these points are not easy to implement, and will require some thought, but currently the analyses are strikingly simplistic and such additions may strongly improve the study.

R1#4 We are confident that our additional analyses, even if simplistic, strongly improved the study. We could not build species distribution models since in some cases, data were either very scarce (rare species), or the GBIF/OBIS data would completely outweigh the data provided by eDNA samples. For instance, the shortfin Mako (*Isurus oxyrinchus*), has more than 8,500 occurrences in GBIF/OBIS, and 3 eDNA detections in our samples, with one of these being an ecological range completion. Building a distribution model including eDNA samples would be useless for such species, as the new occurrences would be treated as outliers and would have little to no impact on estimated niches. We mentioned this as a perspective in the discussion L349:

[Although a limited quantity of eDNA samples collected over a short time period is sufficient to demonstrate biases and to pinpoint the crucial need for more data, it does not allow an effective integration into SDMs. For example, the shortfin mako (*Isurus oxyrinchus*) has more than 8,500 occurrences reported in GBIF/OBIS, while we detected the species only three times with eDNA, one of them corresponding to a range completion. A novel species distribution model just adding some new eDNA occurrences would have little to no impact on the estimated species niche in most modeling frameworks, especially if these occurrences represent ecological outliers.]

Overall, we have requalified the take-home message and clarified our points, and we have added null and logistic regression models to provide more quantitative perspectives in the paper.

**Reviewer #2:**

The article "The uncharted geographic and niche boundaries of marine fishes" written by Sanchez et al combine eDNA based monitoring, species distribution models and GBIF/OBIS species records to investigate the geographic ranges and niches of marine fishes. The authors found geographic range expansions for 93% of the investigated species and niche range expansions for 7% of these species.

Overall, this is a well written article, with however minor misspellings and wording that remains to be corrected. It is interesting to see how the authors combined molecular based detection with SDMs and GBIF/OBIS records to get a better understanding of the marine fish geographic biodiversity. This study will be very useful for better reporting fish diversity across the globe.

While this is a valuable contribution to field, I have several concerns with the manuscript in its current form.

Please find below a breakdown of comments/suggestion to help improving the manuscript.

We would like to thank the reviewer for his/her positive evaluation, and we have addressed his/her comments at our best. As an introductory comment, we would like to mention that we replaced all terms “range expansions” by “range completions” to avoid any confusion with temporal modifications of species ranges.

R2#1 Line 24: what do you mean by "anthropogenic preferences"?

R2#1 We agree that “preferences” was not clear, and we replaced it by “constraints”.

R2#2 Line 32: "in locations previously sampled through classical methods"

R2#2 We modified the sentence accordingly.

R2#3 Line 39: changes

R2#3 We modified the sentence accordingly.

R2#4 Line 57: challenges

R2#4 We modified the sentence accordingly.

R2#5 Line 84: what about DNA transport and persistence (especially DNA transport through predators)? This can greatly impact the spatial and temporal signature

R2#5 We understand the reviewer’s concerns about eDNA transport and signature extent. However, the reference that we use on this line, Murakami *et al.* (2019), shows that eDNA from caged species in seawater was not detectable 2 hours after removing the fish, and in very low concentrations when sampling at 1,000m from the cage. There is increasing consensus in the scientific literature that environmental DNA metabarcoding can differentiate fish communities among close habitats (West *et al.*, 2020; Rozanski *et al.*, 2022; Shea & Boehm, 2024), so the signature is narrower in space and time than previously thought. We added these references (L83).

Given the fact that we work with 10 x 10 km cells, the probability for eDNA to be transported towards another cell more than 10 km away from the source seems minimal. Furthermore, in the case a species would be detected in an adjacent cell from the source of eDNA, it would not result in a large range completion anyway. In case eDNA would be transported through predators, we found in the literature that piscivorous fish digestion and excretion can last a few hours (5 to 7; Juanes & Conover, 1994), or sometimes days in cooler waters (Hop & Tonn, 1998). In the first case, we do not expect a predator to migrate over large distances during this short time period. In the second case, we do not expect eDNA to be excreted in a detectable state after days of digestion, given the fact that caged fish eDNA was not detected 2 hours after removing fish from the cage.

Finally, prey DNA from fish excreta is expected to be less abundant than predator DNA or even the DNA of other species present in the sampling location. Deagle *et al*. (2006) demonstrated using qPCR that prey DNA was approximately ten times less abundant than predator DNA when analyzing a fragment of around 60 bp (similar in size to the *teleo* marker). Species present in the environment, even the rarest ones, continuously release DNA into their surroundings. As a result, the proportion of prey DNA in water samples is likely to be negligible, and the probability of collecting samples precisely at the moment of fish excretion is very low.

R2#6 Line 97-98: this is a bit confusing. What do you mean by this?

R2#6 We agree that this part needs some clarification. We modified the sentence L98 that now reads:

[To only consider species with accurate assignations, and avoid false positives, we removed species from genera in which fewer than 95% of species had been sequenced in neighbouring regions, reducing the dataset to 478 species (see Methods).]

R2#7 Lines 110-111: using a 10x10 resolution is rather small and could artificially lead to an expansion range of most species investigated. What would happen with a 50x50? Would you still have a similar number of species showing an expansion range? 10x10 is also increasing the probability to have a mismatch between eDNA detection and GBIF/OBIS estimates. I understand that using 10x10 is better to increase the resolution of species presence, but I'm wondering if this is not leading to biases in this instance.

R2#7 We also ran the analyses for ecological range expansions with a 50x50 km resolution and found the same number of ecological range completions. However, we agree that the potential mismatch mentioned by the reviewer can artificially increase the number of positive distances, so we answer to this issue in the next comment (R2#8)

R2#8 Line 142: how many species show a range expansion up to 10km, 20km, 50km and 50+? That would be valuable to include such details to highlight that the 10x10 resolution is not an issue here.

R2#8 We thank the reviewer for proposing a relevant solution to the issue mentioned in R2#7.

We found these numbers of range completions for each of the below scales:

440/478 → 10km (93%)

406/478 → 20km (84%)

352/478 → 50km (73%)

297/478 → 100km (62%)

231/478 → 200km (48%)

We included these values for the 20 x 20 km and 50 x 50 km resolutions in the manuscript L155:

[The distribution of the range completions (S1 Figure) showed that they were not the result of an arbitrary choice of a cell size, since 84% of them were above 20 km, and 73% above 50 km.]

R2#9 Lines 146-170: how confident are you with the taxonomic identification step here? This is quite an astonishing expansion for some species. Do you think such short amplicons are reliable enough to differentiate at the species level and avoid false positive records? Could such results be due to the amplification of unknown or closely related species?

R2#9 Since we removed all species detections belonging to genera in which less than 95% of the species pool had not been sequenced (reducing the number of species from 1,225 to 478 species), we have a strong confidence in not having amplified closely related species that had been sequenced. We do mention in the discussion that there might have been false positive detections due to unknown species that have never been sequenced. However, we quantified the number of unique sequences shared between separate genera: 467 out of 19,777 unique sequences (2.3%), in the first paragraph of the results L96. As a consequence, the highest risk of detecting a false positive that persists in this analysis would be due to unknown species pertaining to known genera, specifically in recent-diverging lineages. This potential issue has been discussed L318.

Regarding the short length of the *teleo* marker, allowing the detection of rare species and degraded DNA fragments (Valentini *et al*., 2016; Polanco *et al.*, 2016; Collins *et al.*, 2019), we are not so surprised to find wide range completions. However, longer fragments would allow a better resolution in species identification, to the detriment of eDNA detectability. We mention in the discussion how a multi-marker approach could bring more clues in future studies, specifically adding a longer marker than *teleo,* to confirm some occurrences. We added the aforementioned references in the manuscript L334.

R2#10 Lines 227-232: very interesting! Provide additional details regarding how many species show a range expansion at 10km, 20km, 50km and 50+ would help to better understand the results and implications of the manuscript.

R2#10 We thank the reviewer for this comment, and we have provided this information in response to R2#8.

R2#11 Line 273-276: indeed! Really good to mention.

R2#11 We thank the reviewer for this comment.

R2#12 Line 283: indeed, the filtering steps were quite strong, so it is likely that true positives have been discarded here.

R2#12 We thank the reviewer for this comment.

R2#13 Lines 286-289: indeed. Could be nice to also add a couple of references highlighting the benefits of such a multi primers approach.

R2#13 We respond to this comment in R2#9.

R2#14 Line 293-298: Large volume of filtered water is indeed helpful to maximize eDNA recovery but can lead to inhibition issues. Has it been tested in this study. The large number of PCR replicates help to mitigate false negative detection, but the use of higher natural replicates would have a stronger impact to maximize species recovery. It would be good to mention it.

R2#14 In the current literature, we mostly found publications that promote filtering large volumes of water to maximize species detections (Turner *et al.*, 2014; Thomas *et al.*, 2018; Muha *et al.*, 2019; Sepulveda *et al.*, 2019; Schabacker *et al.*, 2020; Govindarajan *et al.*, 2022; Stauffer *et al.*, 2021). We modified one of our sentences to put the emphasis on the use of more field replicates L341:

[Another reason why we certainly underestimated species occurrences in our eDNA surveys is that we usually sampled around 30 L of seawater in duplicates, so we may have missed a large portion of fish communities since our eDNA sampling effort is relatively low, pointing out that adapted sampling strategies, including more field replicates, are required if eDNA is used (54).]

R2#15 Line 322: "higher volume in some Arctic samples."

R2#15 We thank the reviewer for pointing this out and have modified this sentence.

R2#16 Line 322-323: do you think the contamination was linked to the niskin bottle only? Or was it during downstream processes in the lab?

R2#16 Good point: we detected some contaminations on two different sampling events in which Niskin bottles were used, so we preferred to avoid including this dataset. The contaminant species were detected only in samples collected with Niskin bottles and in none of the dozen samples analyzed in parallel in the same DNA extraction batch or in the PCR amplification batch, neither in the same sequencing batch. eDNA analysis of samples from the geographic region where these species are present had been conducted several months before the samples in which their detections were considered false positives. Among the several hundred samples analyzed between these two batches of analysis, these species were never detected. To rule out any possibility of laboratory contamination, the eDNA analysis was repeated on the contaminated samples, yielding identical results. Additionally, none of these species were found in DNA extractions or PCR negative controls, further confirming that the contamination did not originate from laboratory processes. The samples were collected using a peristaltic pump, which does not allow water to pass through it. The sampling kits were sterile and single-use, eliminating them as potential contamination sources. Given these factors, the only remaining possible source of contamination is the Niskin bottle.

R2#17 Line 324: can you provide details regarding this protocol? Maybe better to rephrase it as well as "a strict contamination protocol" is slightly misleading.

R2#17 We rephrased the sentence to explain how we avoided contamination L371. Please see how the answer to R2#19 provides more information on this matter.

[Filtration was performed using single-use materials, and the pump never came into contact with the samples. All surfaces used for kit preparation prior to sampling were decontaminated with bleach, and collectors wore disposable gloves, changing them between samples. DNA extraction, amplification, and sequencing were conducted in separate, dedicated rooms equipped with positive air pressure, UV treatment, and frequent air renewal. All surfaces were bleached before and after each process, and laboratory technicians wore face masks, disposable full-body suits, hair covers and two pairs of disposable gloves (32). Negative controls were carried out during some fieldwork and in all laboratory operations.]

R2#18 Lines 327-329: it would be good to provide additional details in the manuscript or in SI.

R2#18 We consider that describing the whole protocol in this paper might considerably increase the length of the paper, so we added all the details in supporting information.

R2#19 Lines 345-346: did you removed all sequences present in the PCR blanks? Did you used a read number or RRA%? Can you provide additional details?

R2#19 We modified the sentence L396, that now indicates that the only species found in the negative controls were human and/or domestic animals. Also, we removed sequences that appeared fewer than 10 times as indicated L387, and with a relative abundance below 0.1% as indicated L394.

R2#20 Line 350: how relevant it is to use a database with regional species knowledge when demonstrating a range expansion of 93% of the investigated species? Could this lead to biases in the end results? Do you think using a global database without any knowledge of species distribution would lead to different results?

R2#20 In order to retain assignations with a high degree of confidence, we removed species pertaining to genera with a sequencing coverage under 95%. We computed this sequencing coverage for each detection by recovering the species list of the province it was found in, and the neighbouring provinces. Provinces are defined at a larger scale than ecoregions, so we are already using a very stringent filter in our analyses. Using a global database and removing all genera with a global sequencing coverage under 95% would certainly lead to a drop in the number of species being included in the analysis and prevent us from detecting potential true positive detections. Cryptobenthic species for example, share genera among very distant regions, and would disappear from the analysis if one region had a low regional sequencing coverage, although some regions could reach a full sequencing coverage, preventing us to detect any range completions for these species.

R2#21 Line 378: can you provide the name of the database?

R2#21 We agree that a database should be named. However, this data comes from a data paper (Coro *et al.*, 2018) so we modified the sentence by using “dataset” instead L426.

R2#22 Line 417-…: this is an impressive number of projects mobilized for this study!

R2#22 We thank the reviewer for his/her comments and beneficial additions to the manuscript.

**Reviewer #3:**

This paper claims to have found "expansion" of species geographic ranges and environmental niche using eDNA. However, it does not do that. No temporal trends are shown at all.

We apologize for the misunderstanding. We replaced all terms “range expansions” by “range completions” in order to avoid any confusion with temporal modifications of species ranges.

R3#1 What it has done is collect more samples using eDNA to document the occurrence of fish. The results could simply be due to further sampling, not necessarily the use of eDNA. There is no statistical analysis to test whether it is their method, or just more sampling, that explains the new geographic records.

R3#1 We thank the reviewer for this remark, and we have answered this issue in R1#2, with the use of a null model:

[R1#2 Thank you for the suggestion. We set up a null model to test if our eDNA samples, whether they address a methodological or a geographic bias, contribute to ecological range completions beyond what would be expected from adding new GBIF/OBIS occurrences randomly. In other words, we tested whether species range completions were due to a sampling effect, *i.e.* adding a certain number of samples, or to new sampling effort towards certain overlooked locations combined to eDNA methodology.

We generated 1,000 samples for each species, that were randomly selected among their total number of GBIF/OBIS occurrences. More precisely, we randomly selected a subset by removing a number of GBIF/OBIS occurrences (*N*), where *N* corresponds to the number of new eDNA occurrences for a given species. For each of these random samples, we then computed the ecological range completion after adding the *N* randomly removed GBIF/OBIS occurrences. This produced a null distribution of expected range completions under the assumption that adding *N* new random occurrences (without our eDNA samplings) changes the perceived species ranges. We then compared our observed range completion (calculated when adding our eDNA occurrences) to this null distribution. We found that for 31 out of 62 range completions, the observed completion was in the top 5% of the expected values. This suggests that observed range completions with eDNA samples were significantly larger than expected from random sampling GBIF/OBIS occurrences alone. The remaining 31 completions fell within the expected range, meaning they could be the result of simply adding new samples.

However, a key limitation of our null model is that it only generates random occurrences within previously sampled locations. Since our eDNA samples occur in locations where no data was available, the model cannot generate random occurrences in these unsampled GBIF/OBIS locations. As a result, when eDNA contributes to range completions in previously unsampled regions, we cannot determine whether this effect is due to eDNA’s unique ability to detect species or simply because we sampled a new overlooked area. We included this new important analysis in the results and the methods.]

R3#2 Neither do they report the dates of past records, as it could be that if some are decades old then species range extensions could be due to climate change. Many studies have shown range extensions of fish correlated with climate warming. Were the dates when species were recorded in OBIS, GBIF or by eDNA matched to the environmental conditions at that place and time?

R3#2 We do mention in the discussion that our framework does not allow the distinction between climate range shifts and ecological range completions; what we clearly pinpoint is the absence of data for a given species in a given cell. Including old records helps prevent misinterpreting our recent data as evidence of range completions when, in reality, the species had already been recorded in those locations in the past. This is especially important in remote regions where historical sampling was sparse.

All cells were matched with a single median value of recent monthly data gathered from 2017 to 2021, as indicated in the methods L426. Since most records are recent, as indicated in the methods L112, the niche boundaries have a relatively low probability to be biased, and if our eDNA detections occur in cold waters (ex: shortfin mako in the Canadian Arctic), these cold waters correspond to recent, warmer dates since we gathered the environmental data from 2017 to 2021, reducing our chances to detect the species in an excessively cold water.

Nonetheless, we may have detected some species range shifts that happened during an extreme event that can, in turn, trigger a colonization event. We further discuss this issue L341:

[Some of the reported completions could also actually be range shifts but our framework does not allow us to separate range shifts and range completions. Such range shifts may happen during extreme events, trigger a new colonization event, and so need to be documented (54).]

R3#3 The introduction suggests the paper will use Species Distribution Models but it never does. Determining the species' environmental niches seems to be based only on the environment on where species were recorded, without accounting for sampling effort as SDM can help with.

R3#3 We understand that it may be confusing to mention SDMs in the introduction for the reader, without using them afterwards. We included a sentence in the introduction to explain that our analyses do not rely on SDMs L89.

[Here, we use over 6 years of eDNA surveys and nearly one thousand samples across the world’s oceans, including polar regions and tropical remote islands, to reveal sampling biases in species geographic ranges, as well as in environmental and anthropogenic ranges (hereafter ecological niche ranges). Rather than using SDMs with limited additional occurrences, we compare species geographic and niche ranges without and with the new occurrences, and employ a null model approach to evaluate the benefits of sampling new locations and using eDNA metabarcoding for the completion of geographic and niche ranges.]

We also clarified with the help of Fig. 2 the fact that we only used the minimum and maximum values of environmental and anthropogenic niches at which the species was observed rather than determining the niche as a mathematical function.

R3#4 It is not novel to say there is geographic undersampling of species. This has always been the case and probably always will be. One cannot accuse databases of being biased if they simply report what has been submitted to them (lines 211), just as one would not accuse authors of bias in a paper when they do not report what they did not do or find. I appreciate this misuse of language may be because none of the authors are native English speakers but there are many authors with a lot of experience. I wonder if because there are so many that none of them have paid much attention to the quality of the writing of the paper.

R3# Our objective was not to accuse databases of being biased, but rather to try to show and quantify this bias. We have carefully modified all sentences that might look like an accusation against existing databases. Furthermore, while it is recognized that certain geographical regions are under-sampled, there is, to our knowledge, no quantification in the literature of how this affects our understanding of species distributions and niches and how eDNA surveys may help to fill these gaps.

R3#5 Other aspects of the paper also indicate confused thinking and interpretation. e.g., "human impacts" is not part of the environmental niche of a species. (line 52, 189)

R3#5 We do not include humans in the environmental niche, and this is why we specifically use the term “ecological niche” throughout the manuscript (see R3#13). Based on recent literature (Feng *et al.*, 2024; Frans & Liu, 2024), humans have an influence on how species are distributed, and some anthropogenic drivers can be treated as novel dimensions of species ecological niches. We follow this integrated aspect of the niche in the Anthropocene.

R3#6 They seem to misunderstand the nature of the GBIF and OBIS databases. These publish datasets. They do not "report" (line 59) or "assess" (line 212) anything. To accuse them of this would be analogous to claiming a scientific journal (which only publishes papers submitted to it), was underreporting or assessing something.

R3#6 We modified these sentences to avoid any misunderstanding, L63:

[Meanwhile, data aggregated in GBIF and OBIS often fail to capture the full extent of biodiversity, particularly in the tropics where half of the cryptobenthic reef fishes remain missed (10,16).]

and L239:

[there is still a significant bias in the assessment of marine fish biodiversity based on existing global occurrence databases such as GBIF and OBIS.]

R3#7 One should not cite OBIS for a specific record in a locality but rather the source dataset (line 158).

R3#7 For GBIF records, you may find in the references the DOI linking to a filtered export mentioning all datasets used in the analyses. For OBIS, their team is currently working on creating such DOI for us, and we are still waiting for it.

R3#8 Similarly, it is unreasonable to accuse publishers (OBIS, GBIF) of "missing" to sample something (line 226) when they do not do any sampling.

R3#8 We replaced “missed by GBIF/OBIS” with “missed in GBIF/OBIS” to avoid any misunderstanding.

R3#9 The understanding of the literature is questionable. The paper cited for species being introduced through the Panama canal only raises it as a concern if the canal salinity increases. But at present it is freshwater and marine species are unlikely to survive. There does not seem to be any evidence of marine species being introduced to either the Pacific or Caribbean via the Panama canal. However, is it possible that the DNA of a fish survived the transport through the canal? (line 163). It seems the authors assume the presence of DNA means a living animal was present but it is well known that DNA can survive a long time (even in sediment cores). This potential transport of DNA does not seem to have been considered anywhere in the interpretation of the results. If feasible, it significantly undermines the premise of the results indicating geographic or environmental range extensions of the species.

R3#9 This topic has been discussed many times in the literature, and we have mentioned Murakami *et al.* (2019) to support our findings. Please, see R2#5 where we answer a similar question.

R3#10 The paper claims that there have been no comparisons between fish species recorded by conventional and eDNA sampling methods ("not documented" lines 85-87) but I believe there have, including by some of the present authors. e.g., <https://nsojournals.onlinelibrary.wiley.com/doi/full/10.1111/ecog.06299>; <https://onlinelibrary.wiley.com/doi/full/10.1002/edn3.140>; <https://royalsocietypublishing.org/doi/10.1098/rspb.2020.0248>; This suggests they are being a bit disingenuous about the novelty of the paper.

R3#10 We do not claim that no comparisons were ever made, but rather that :

[the extent to which eDNA surveys can complement classical surveys and global databases of marine fish occurrences in undersampled regions and ecological conditions to ultimately unveil unsuspected geographic and ecological ranges niche distributions has not been documented.]

R3#11 Figure 1 (a) and Figure 2 blend dark blue into black and so fail to show the patterns suggested. They indicate most of the ocean is sampled to maximum intensity (Fig 1a). This is so bad it is disappointing the paper was even submitted and sent out for review. Surely with so many authors some could have spotted this problem?

R3#11 In Figure 1a, we believe that the reviewer missed that maximum intensity was purple, whereas the background color is darker. To avoid any confusion, we changed the maximum intensity to a clearer color, and the background to black. In Figure 2, there is no background, the darkest colours correspond to the largest distances, as explained in the caption.

R3#12 Note that the licence to use GBIF and OBIS databases is CC-BY per dataset; but none of the datasets are cited. Thus the authors are in breach of the licence conditions. This is like citing a journal without indicating which papers were used.

R3#12 The licence for GBIF and OBIS can also be used with a filtered export and a corresponding DOI . We failed to include an OBIS DOI, which is now being produced by the OBIS support, thanks to the reviewer’s comment.

R3#13 The language is also loose or mistaken in places. e.g.,

Line 43: what are "social-environmental variables" (also line 134)

Line 211: what "extensive sampling campaigns conducted across all oceans" have there been. for in situ sampling this is impossible (perhaps for satellites).

The paper is generally poorly written. The Introduction repeats points in different paragraphs and could be much shorter. The Results mix in Discussion as indicated by references to other literature cited.

R3#13

We thank the reviewer for pointing this out, we replaced every occurrence of “social-environmental” by “ecological”, while highlighting in the introduction that this includes anthropogenic factors.

We thank the reviewer and have modified the sentence L238:

[Despite the extensive sampling efforts made during the last decades, which together encompass all oceans, …]

R3#14 While EDNA is a valuable new sampling method, every method has its biases. Thus it is not "accurately" assessing (line 81) fish occurrences for reasons the authors later note.

R3#14 We removed the word “accurately”.
