# Supplementary material for: eDNA surveys substantially expand known geographic and ecological niche boundaries of marine fishes
Source: PLoS Biol. 2025 Oct 30;23(10):e3003432. doi: 10.1371/journal.pbio.3003432 (PMC12574855; doi:10.1371/journal.pbio.3003432)
Supplement: Attachment — Submitted filename: Response to reviewers v3_final.docx [file pbio.3003432.s011.docx]

**Reviewer #1:**

[identifies himself as Jonathan Belmaker]

This study uses a comprehensive and impressive global eDNA sampling campaign to show that the current environmental and geographical niche, as estimated by GBIF and OBIS, is underestimated for a vast majority of species. The actual results are not surprising, as biases in such GBIF and OBIS occurrences records are well documented. However, the sheer scale of the analyses presented here make this a strong case for the need for more, and better, sampling. I commented on the original version of the manuscript and find that most of my comments were addressed to a certain degree. I like the use of the logistic regression and null model to try and gain deeper insights.

R1#1 However, I still feel the study falls a bit short in using the new eDNA data to say some more quantitate about how to select sites for sampling and what method to use. I think some more practical advice can go a long way. The authors replied to my previous comment by writing that estimating the time and funding efforts between methods seems out of the scope and could be the goal of a separate paper. I agree to some extent, but without something more concrete the take-home message is just saying we are undersampling nature and that our samples are biased in multiple ways (this is most evident in the end of the abstract). However, we already know that and as a reader I would be much happier to feel I have learned something new.

R1#1 We understand the need, as a reader, for information on the actual benefits of eDNA. We thank the reviewer for pushing this point further and making us rethink this aspect with complementary analyses. In response we have computed a new model, based on the 275 cells where our eDNA surveys were carried out. We modeled the number of new species gained in a cell, using Boosted Regression Trees, as a function of (1) eDNA sampling effort, (2) GBIF sampling effort, (3) GBIF richness, and (4) environmental factors. The model provided a correlation between observed and predicted values of 0.96, while the correlation on a tenfold cross-validation was 0.83.

We used this accurate model to predict the number of species gained if 10 eDNA samples were added in each grid cell, and represented these predictions on a global map (new Fig. 2 in the revised paper). We added a description of the results in the revised version L149 and discussed it L325. We also included the partial dependence plots in Supplementary Information. Moreover, this allowed us to obtain an average price per newly detected species.

We hope this additional analysis and the new Figure fulfill your requirement.

*Figure 2: Map of the predicted number of fish species gained in a cell if 10 eDNA samples were added to GBIF/OBIS sampling records, if any. Predictions obtained from a Boosted Regression Tree using the eDNA sampling effort, the sampling effort estimated from GBIF/OBIS, the species richness obtained from GBIF/OBIS, bathymetry, Sea Surface Temperature, Net Primary Productivity, Sea Bottom Dissolved Oxygen, Sea Bottom Temperature, and the Cumulated Human Impact as predictors across the 275 eDNA sampled cells in this study.*

[In order to assess the benefits of eDNA surveys, we used Boosted Regression Trees (BRT) to model the number of species gained per cell as a function of the eDNA sampling effort, the sampling effort estimated from GBIF/OBIS, the species richness obtained from GBIF/OBIS, bathymetry, and the environmental and anthropogenic variables across the 275 eDNA sampled cells in this study. The BRT model showed very high predictive power, with a correlation of 0.96 between predicted and observed values, and a correlation of 0.83 based on a 10-fold cross-validation. Partial dependence plots highlighted that the number of species gained (over 18) plateaued after 10 eDNA samples (S1 Figure). In contrast, species gains decreased sharply with the increasing GBIF/OBIS sampling effort, reaching its minimum at around 130 GBIF/OBIS sampling records (S1 Figure). We then generated predictions with this accurate BRT model to estimate how many additional species 10 eDNA samples would yield in each cell: we found a mean gain of 23 (s.d.± 14.52) species and a maximal gain of 98 species in undersampled, remote tropical areas (Fig. 2). In our study, the cost of analyzing 10 eDNA samples corresponds to approximately 5,000 euros, excluding the cost of transport to the sampling point. So, on average, the detection cost of a new species is worth 277 euros but is highly variable across regions.]

R1#2 I also have a major comment on the implementation of the null model. The null model is based on randomly removed GBIF/OBIS occurrences. However, as these are aggregated in space they will a-priori represent a limited geographic and environmental space compared to the eDNA samples. A more revealing null model will be one that mimics the geographically dispersed sampling of the eDNA data. This may require some thought.

Reminder of the comment in the last round of review:

*The authors acknowledge that niche expansion estimates will depend on the number of occurrences (for under sampled species each new occurrence will likely expand the niche). However, this is never actually accounted for or quantified. Here too, I feel the use of some kind of null model to show how much each new eDNA occurrence contributes to niche expansion beyond what would be expected from another occurrence for that particular species (potentially separately estimated within or outside the estimates species range, e.g., using a convex hull) will make this manuscript a lot stronger.*

Here, we understand that the reviewer’s main concern is the effect of the number of occurrences of each species on the probability of expanding the niche. In the first revision we proposed two new analyses to address R1’s comments: logistic model (to address the lack of quantitative analyses) and a null model (to address the lack of analysis on the number of species occurrences). We acknowledge that this was not necessarily the best way to respond to the reviewer’s concerns. Indeed, the logistic regression included in Supporting Figure 2 not only provides a more quantitative aspect, but most importantly shows the relationship between the number of occurrences and the probability of new eDNA detections to expand a) the geographic range, and b) the ecological niche range.

During the second revision phase, we built another null model that mimics our dispersed eDNA sampling design by randomly adding new occurrences to cells that were sampled by eDNA, with a sampling probability in each cell equal to the occurrence probability of each species in each cell (obtained from co-occurrence patterns, using a method similar to that of Dark Diversity; Carmona & Pärtel, 2020). The issue with this null model framework, is that our eDNA sampling was not random at all, and we have a lot of samples from polar regions, which means that many of the randomly selected samples fell in polar cells, dramatically and artificially increasing the niches of nearly all species.

We decided to rely on the logistic regressions to provide a clear relationship between detection probability of a given species in a given cell as a function GBIF sampling effort, the number of occurrences and the species body size. To strengthen this analysis, we included more descriptions of the explanatory power (AUC) of both models, and the order of importance of the variables L267. We also kept the original null model as it does provide complementary information that we can discuss. Together these models suggest that (1) the probability to detect range fillings is primarily shaped by the sampling effort, and the number of occurrences, and (2) that only half of ecological niche range fillings would be due to eDNA surveys, beyond a random allocation of classical sampling effort.

**Minutia:**

R1#3 The second sentence of the abstract is just too long.

R1#3 We have split that sentence into two parts. See L29.

R1#4 Line 34: Instead of "whether" (the answer is trivially yes) ask to what extent / where.

R1#4 We have modified that sentence accordingly L34:

[Here, we used nearly one thousand environmental DNA (eDNA) samples across the world’s oceans, including polar regions and tropical remote islands, to determine the extent to which geographic and ecological niche ranges of marine fishes are underestimated through the lens of GBIF and OBIS occurrence records.]

R1#5 Line 35: "We completed known geographic ranges" . It is not clear what you mean here before reading the rest of the ms.

R1#5 We have modified the sentence accordingly:

[Our sampling revealed that the known geographic ranges for 93% of species and the ecological niche ranges for 7% of species were underestimated, thereby filling them.]

R1#6 Lines 38-40: This is a trivial statement. Can a more original conclusion be reached?

R1#6 We have modified the final sentence of the abstract according to the results now included in the revised paper L41.

[Using a machine learning model, we found that a local effort of 10 eDNA samples would detect 24 additional fish species on average and a maximum of 99 species in previously unsampled tropical areas. Yet, a null model revealed that only half of ecological niche range fillings would be due to eDNA surveys, beyond a random allocation of classical sampling effort. Altogether, our results suggest that sampling in remote areas and performing eDNA surveys in over-sampled areas may both increase fish ecological niche ranges towards unexpected values with consequences in biodiversity modeling, management, and conservation.]

R1#7 Line 54: "without assessing their spatiotemporal congruence" - not clear what you are trying to say here.

R1#7 We removed this part since it was confusing.

R1#8 Lines 55-58: This sentence on anthropogenic factors and SDMs is disjunct and not really relevant to the topic at hand.

R1#8 We agree and have split this paragraph into two parts, and merged them with the two paragraphs immediately before and after. See L60.

R1#9 Line 66: explain what GBIF and OBIS are.

R1#9 Indeed, we forgot to add (GBIF) and (OBIS) to the previous paragraph after mentioning them. We have now added this information, L70.

R1#10 Lines 73-76: This sentence is unclear. We are dealing with the difficulty of detecting small species in the open ocean. How are these issues (dispersal, key support to people) relevant?

R1#10 We actually meant that it is even more critical to estimate niches and geographic distributions of these species, but indeed dispersal is not relevant in this sentence. It now reads:

[For fish, this estimation is even more critical since they are sensitive to both environmental and anthropogenic pressures (*e.g.,* habitat degradation) (10), and play important roles in linking marine ecosystem functioning and human societies (19), as well as supporting key contributions to people (20).]

R1#11 Lines 137-138: Geographic and ecological niche range completions need a definition / explanation.

R1#11 We have included a better description of Figure 3 in the main text, to explicitly define what geographic and ecological niche range fillings are, L172.

[Aggregating all these data with our eDNA samples allowed us to reveal geographic and ecological niche range fillings (Fig. 3), the former being measured as the distance between a new detection and its nearest GBIF/OBIS occurrence, and the latter being the relative increase in the niche breadth.]

R1#12 Lines 231-233 and 300-304: "some of the completions we found may only be the result of a sampling effect." - all completions are a result of limited sampling. By definition. What you mean is explained later on, but here it is not clear.

R1#12 We have modified all these sentences 279, L283 and L362, replacing “due to sampling effect” by “due to the low number of species occurrences”.

**Reviewer #3**

The novelty in this study is that by using a very impressive collection of DNA samples of fish from around the world, the authors show how DNA can pick up species not sampled by other methods and show several species are more widespread than previously reported. A more in-depth examination of these range extensions would be worthwhile and of great interest to fish biologists and biogeographers.

R3#1 Unfortunately, the authors try to spin the story into something it is not. In the first version they claimed they discovered range expansions when they did not; they only reported new locations for species. Now they claim their work "completes" knowledge of species ranges. This is similarly incorrect. There is no doubt that more data will continue to fill gaps in our knowledge of species distributions. Thus the paper based on its present conclusions and claims has to be rejected.

R3#1 We have replaced “range completions” by “range fillings”.

R3#2 Going forward, the authors have a valuable data set and insights and should focus on those. If time allowed they could match how well published records in OBIS and GBIF fill the predicted ranges of species available in AquaMAps, IUCN Red List, OBIS (has a new range map platform) or AquaX. They could say with more confidence how their data has "completed" gaps in species geographic ranges.

R3#2 We appreciate the reviewer’s suggestion; however, we believe it falls outside the scope of the current paper. Nonetheless, we are confident that the data provided in this work will contribute meaningfully to further advancements in niche modelling.

R3#3 The way the first version was written misled referees into thinking the authors had used SDM, but now it is clear that they did not. It is common knowledge that all sampled data are biased and geographic distributions incomplete. To claim this as a key finding is very weak and undermines the main findings of the study.

R3#3 This comment concords with R1#6. We have modified the final sentence of the abstract according to the results now included in the revised paper. (See line 41)

[Using a machine learning model, we found that a local effort of 10 eDNA samples would detect 24 additional fish species on average and a maximum of 99 species in previously unsampled tropical areas. Yet, a null model revealed that only half of ecological niche range fillings would be due to eDNA surveys, beyond a random allocation of classical sampling effort. Altogether, our results suggest that sampling in remote areas and performing eDNA surveys in over-sampled areas may both increase fish ecological niche ranges towards unexpected values with consequences in biodiversity modeling, management, and conservation.]

R3#4 It is not clear what data they used from OBIS and GBIF, not only because they do not list the datasets used, but they seem to suggest they only used data from samples collected from 2017-2021 (stated in response to referees, or 2023 on line 370).

R3#4 We agree that our subtitles in the methods section were not clear. We have now changed them to simplify the Methods section.

However, it is clear that we refer to eDNA LXXX (and thus 2017-2023, not 2017-2021 as stated by the reviewer). The OBIS and GBIF data are described in the next section, L483. The dates are also clearly explained, as stated in the next reviewer’s comment (R3#5).

R3#5 Why? Why not use all records from an area in OBIS and GBIF. But then on lines 427 they say they downloaded OBIS data from 1742 to 2023, but GBIF only 2006 to 2023. So it is unclear what data were used for comparison. This means their whole comparison is biased because they disregarded another 100 years or more of data in these databases.

R3#5 We mentioned that we used both OBIS (1742 to 2023) and GBIF (2006 to 2023, for computational time issues, as the downloads take a very, very long time to obtain). Moreover, OBIS is a database that focuses on marine species occurrences, so we believe that using this database over a longer time window decreases the likelihood of missing important species.

The reviewer says it is unclear what data we used for comparison: as explicitly mentioned in the paper, we use OBIS and GBIF as data sources. We did not disregard the old occurrences since we include records from years before the 21^st^ century in OBIS. Also, we checked and mentioned that the vast majority of occurrences date from very recent years.

R3#6 Despite a referee's suggestion, the authors refuse to acknowledge that the data in OBIS and GBIF come from distinct datasets which the licence-to-use requires they be cited. A DOI does not do this. The citation can be very simply copied from the metadata downloaded data and added as an Appendix. Scrutiny of these datasets may well find that some come from fishery research trawls which only sample demersal fish over a certain size. Thus, those datasets will miss small gobies, etc However, scuba diving surveys may report them. The paper could then compare DNA methods with other fish sampling METHODS more truly.

R3#6 We clearly mention that we downloaded data for all the species detected during our sampling. We did not choose specific datasets or methods. In this sense, OBIS and GBIF let the user cite a personal DOI that references all the datasets that have been used in a filtered export. We reference the DOI for both filtered exports (references 33 and 34) and invite the reviewer to check these references if needed.

R3#7 The paper is also misleading in claiming to look at ecological niche when it only matches distributions to 3 environmental variables (temperature, productivity, oxygen). USing productivity is an odd choice because this does not affect the biogeographic distribution of fish species; only local and seasonal abundance. There are no ecological variables there, like species associations, habitat, predation or herbivory, etc.

R3#7 We understand that many variables that impact species distributions are not mentioned here: it was a choice to use a limited number of variables, to show that even with a few variables we were able to fill gaps in species niche ranges. Moreover, these variables are those used to estimate environmental preferences in Aquamaps.

R3#8 The use of the ocean index of human impact to define a niche is an interesting idea, but there is no justification for it. No fish distribution is restricted by human impacts (some marine mammals may be). The abundance of some fish is affected by fisheries, but even overfished species can still be common (e.g., cod).

R3#8 We based our work on recent peer-reviewed literature showing that local extirpations exist and that human impact is negatively correlated with species diversity (see refs 9 to 12 in the main text, Mathon *et al.*, 2023; Nikolaou & Katsanevakis, 2023; Feng *et al.*, 2024; Frans & Liu, 2024). Moreover, it is increasingly recognized that human impacts greatly influence species' geographic ranges. In this light, historical distinctions between natural and anthropogenic factors have become blurred, and a coupled human–natural seacape to define species niches is recognized as the new norm.

R3#9 Why the paper declines to add a comment on the relative costs of conventional sampling vs DNA (as recommended by a referee and is a good idea) is not clear. I am sure with some assumptions such a cost estimate would be possible.

R3#9 We agree with the reviewer that we should have included this analysis earlier (see R1#1):

We understand the need, as a reader, for information on the actual benefits of eDNA. We thank the reviewer for pushing this point further and making us rethink this aspect with complementary analyses. In response we have computed a new model, based on the 275 cells where our eDNA surveys were carried out. We modeled the number of new species gained in a cell, using Boosted Regression Trees, as a function of (1) eDNA sampling effort, (2) GBIF sampling effort, (3) GBIF richness, and (4) environmental factors. The model provided a correlation between observed and predicted values of 0.96, while the correlation on a tenfold cross-validation was 0.83.

We used this accurate model to predict the number of species gained if 10 eDNA samples were added in each grid cell, and represented these predictions on a global map (new Fig. 2 in the revised paper). We added a description of the results in the revised version L149 and L325. We also included the partial dependence plots in Supplementary Information. Moreover, this allowed us to obtain an average price per newly detected species.

We hope this additional analysis and the new Figure fulfill your requirement.

*Figure 2: Map of the predicted number of fish species gained in a cell if 10 eDNA samples were added to GBIF/OBIS sampling records, if any. Predictions obtained from a Boosted Regression Tree using the eDNA sampling effort, the sampling effort estimated from GBIF/OBIS, the species richness obtained from GBIF/OBIS, bathymetry, Sea Surface Temperature, Net Primary Productivity, Sea Bottom Dissolved Oxygen, Sea Bottom Temperature, and the Cumulated Human Impact as predictors across the 275 eDNA sampled cells in this study.*

[In order to assess the benefits of eDNA surveys, we used Boosted Regression Trees (BRT) to model the number of species gained per cell as a function of the eDNA sampling effort, the sampling effort estimated from GBIF/OBIS, the species richness obtained from GBIF/OBIS, bathymetry, and the environmental and anthropogenic variables across the 275 eDNA sampled cells in this study. The BRT model showed very high predictive power, with a correlation of 0.96 between predicted and observed values, and a correlation of 0.83 based on a 10-fold cross-validation. Partial dependence plots highlighted that the number of species gained (over 18) plateaued after 10 eDNA samples (S1 Figure). In contrast, species gains decreased sharply with the increasing GBIF/OBIS sampling effort, reaching its minimum at around 130 GBIF/OBIS sampling records (S1 Figure). We then generated predictions with this accurate BRT model to estimate how many additional species 10 eDNA samples would yield in each cell: we found a mean gain of 23 (s.d.± 14.52) species and a maximal gain of 98 species in undersampled, remote tropical areas (Fig. 2). In our study, the cost of analyzing 10 eDNA samples corresponds to approximately 5,000 euros, excluding the cost of transport to the sampling point. So, on average, the detection cost of a new species is worth 277 euros but is highly variable across regions.]

R3#10 Line 56 - human impacts do affect local species distributions, but for fish, not their global. Neither are human impacts part of a species ecological niche (but see above).

R3#10 See R3#8.

R3#11 Line 66 - accusing OBIS and GBIF of "fail to capture the full extent of biodiversity" is misleading and unfair. There is no study or databases that could ever do this, including the present one (claiming "completeness"). Any data availablae depends on it being collected and published. Woudl the authors claim this journal or any other "fails to capture ....". ?

R3#11 We have already modified all the text where it was misleading. Here, we do not accuse OBIS and GBIF to fail, since the subject of the sentence is “the data aggregated” and not OBIS and GBIF L74:

[Meanwhile, the occurrence data aggregated in GBIF and OBIS often fail to capture the full extent of biodiversity, …]

R3#12 Line 88 - this seems to deliberately exaggerate the novelty of the study by adding so many caveats to the sentence. This may be the first "global" comparisons of deteciton of fish by DNA vs tradiotnal methods. But surely regional and local studies are comparable in detectability? Why are their findings not summarised?

R3#12 The scope of this paper is not, and has never been to provide a comparison of detection of species between eDNA and conventional methods. See L96:

[Yet, the extent to which eDNA surveys can complement classical surveys and global databases of marine fish occurrences in undersampled regions and ecological conditions to ultimately unveil unsuspected geographic and niche ranges has not been documented.]

R3#13 Line 188 - this page is the most interesting results; finding species are more widespread is worth exploring further.

R3#13 We understand that this part could be developed with more species but adding more specific cases would relatively extend the length of the paper.

R3#14 Line 275+ - we do not need analysis to tell us that DNA (or any method) is more likely to find an unrecoreded species in a place where there are no recorded species.

R3#14 In the previous submission, L275 we wrote :

[Yet, no study has investigated knowledge gaps at the species scale, considering ecological niche ranges with an alternative sampling approach.

Our eDNA sampling efforts in remote, previously unsampled locations broaden niche conditions beyond the ones that have been extensively sampled these last decades, adding geographical areas and ecological niches hosting unexpected species.]

We do not understand the reviewer’s comment in this sense.

R3#15 Line 261 - claiming the DNA + OBIS + GBIF is now a complete range, and then calculating how much DNA contributed to this "completion" is a circular self-made argument. If they plotted species discovery curves for areas then they may get an idea of completion (e.g., see paper by Mora et al in Proc. R. Soc B some years ago), but that was not done here.

R3#15 Here we believe that R3 has been misled by our erroneous use of “completions”, a word that has been replaced by gap filling throughout the document.

R3#16 Maps in Fig1 (a) and fig 3 blend dark blue into black to it seems the open ocean is the mbest sampled. Also very poor resolution.

R3#16 In Fig. 1, the maximal color of the scale is now clearly purple, so there can be no confusion between high values and the dark background. In Fig. 3, the darkest colors (black/dark blue) correspond exactly to what they’re supposed to : high distances. There is no background in Fig. 3. We provided high resolution images in this submission but the pdf version may have degraded the Figures.
